# Recent Progress in Vaccine Development Against Chikungunya Virus

**DOI:** 10.3389/fmicb.2019.02881

**Published:** 2019-12-19

**Authors:** Shan Gao, Siqi Song, Leiliang Zhang

**Affiliations:** ^1^Institute of Basic Medicine, Shandong First Medical University & Shandong Academy of Medical Sciences, Jinan, China; ^2^School of Basic Medicine, Qingdao University, Qingdao, China

**Keywords:** chikungunya fever, CHIKV, live-attenuated vaccine, VLP, chimeric vaccine

## Abstract

Chikungunya fever (CHIKF) is an acute infectious disease that is mediated by the mosquito-transmitted chikungunya virus (CHIKV). People infected with CHIKV may experience high fever, severe joint pain, skin rash, and headache. In recent years, this disease has become a global public health problem. However, there is no licensed vaccine available for CHIKV. Accumulating research data have provided novel approaches and new directions for the development of CHIKV vaccines. Our review focuses on recent progress in CHIKV vaccine studies. The potential vaccine candidates are classified into seven types: inactivated vaccine, subunit vaccine, live-attenuated vaccine, recombinant virus-vectored vaccine, virus-like particle vaccine, chimeric vaccine, and nucleic acid vaccine. These studies will provide important insights into the future development of CHIKV vaccines.

## Introduction

Chikungunya fever (CHIKF) is a recurrent infectious disease caused by the chikungunya virus (CHIKV). The main clinical symptoms are arthritis and fever. Patients may also suffer from headache, myalgia, and rash. The mortality of CHIKF is below 0.5%, lower than dengue fever, which has similar clinical symptoms. For infants under 1 year old and people over 60 years old, the mortality will significantly increase (Langsjoen et al., 2018). The acute symptoms usually disappear within about 1–2 weeks, but patients may endure long-lasting joint pain and fatigue (Elsinga et al., 2017). Since CHIKV was first isolated in 1952 from Tanzania, it has induced several outbreaks, mainly in Africa and Asia. However, after re-emerging in 2004 in Kenya, the epidemic area expanded from the tropical zone to even Europe and America (Enserink, 2007). The unprecedentedly rapid and wide spread of this disease calls for efficient preventive measures.

**FIGURE 1:**
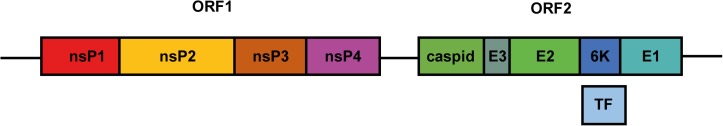
Schematic diagram of the CHIKV genome. The genome of CHIKV is a single-strand RNA compromising two ORFs. ORF1 encodes four nsPs. Translation and further processing of ORF2 produce structural proteins capsid, E3, E2, 6K, and E1. During translation, ribosome shifts to –1 reading frame in 6K, leading to the production of TF protein.

Belonging to the genus Alphavirus of the *Togaviridae* family, CHIKV is an enveloped arthropod-borne virus (arbovirus) with *Aedes aegypti* and *Aedes albopictus* as its primary vectors. The genome of CHIKV consists of a positive-sense RNA approximately 11.5 kb in length. The CHIKV genome comprises two open reading frames (ORFs) that encode four non-structural proteins (nsPs) and one structural polyprotein (Figure 1). The nsPs function as a replicase complex, which not only replicates genomic RNA for progeny but also transcribes subgenomic RNA to express structural proteins (Ljungberg and Liljestrom, 2015). As for the structural polyprotein, it will be further cleaved to capsid and E3-E2-6K/TF-E1 (Figure 1). The latter is important for virion assembly and virus entry. E1/E2 glycoprotein in the envelope was reported to mediate cell binding at the early stage of infection (Strauss and Strauss, 1994).

The large-scale resurgence of CHIKV is, to some extent, due to social and economic developments, such as the increased number of overseas tourists, the high population density brought about by urbanization, and the changes in mosquito distribution caused by global warming. A lot of antiviral compounds have shown valuable therapeutic efficacies, especially during CHIKF outbreak. Since it is one of the most cost-benefit public strategies to prevent infectious disease, vaccine is an indispensable means for preventing CHIKF. Considering that the CHIKV antigen variety is limited and infection may lead to lifelong immunity, the advantage of vaccination is particularly prominent.

The attempt to develop a CHIKV vaccine started from the 1960s, not long after the virus was isolated. Since then, researchers have continued to develop CHIKV vaccine candidates that balance immunogenicity and safety. However, there is no licensed CHIKV vaccine available for use. Researchers have taken advantage of progress in biochemical and molecular methods and have utilized various strategies to develop vaccines, which can be classified as inactivated viral vaccine, subunit vaccine, live-attenuated virus (LAV) vaccine, recombinant virus-vectored vaccine, chimeric vaccine, virus-like particle (VLP) vaccine, and nucleic acid vaccine. In the majority of this review, we focus on novel CHIKV vaccine development and progress in the evaluation of vaccine candidates since 2016.

## Inactivated Vaccine

The first attempts to develop a CHIKV vaccine emerged shortly after the first CHIKF outbreak in the 1960s. Early studies adopted inactivated vaccine as the preferred strategy. By inactivating the virus via heating or chemical treatment (formalin), researchers generated vaccines that could stimulate the immune response without risk of infection, which conferred inactivated vaccine with high safety.

Researchers first infected mouse brains with an African genotype strain of CHIKV and successfully collected neutralizing antibodies 15 days post infection (Kitaoka, 1967). The most prominent achievements in early CHIKV vaccine development were made at the Walter Reed Army Institute of Research, based on a series of platforms including chicken embryos, suckling-mouse brains, and African green monkey kidney cells. The first evaluation of inactivated vaccine in humans was reported in 1971 (Harrison et al., 1971). Two groups of healthy volunteers were vaccinated twice (day 0 and 28) with 0.5 or 1 mL, respectively. Both groups developed neutralizing antibodies within 2 weeks without adverse effects.

In the following 40 years, many vaccine candidates based on inactivation have been developed and have entered the clinical phase. One inactivated vaccine, which was produced in Vero cells, stimulated both cellular and humoral immune responses, with the peak titer of neutralizing antibodies appearing at 6–8 weeks post-vaccination (Tiwari et al., 2009). Kumar et al. (2012) evaluated the protective efficacy of an E2 protein-based recombinant vaccine and whole-virus inactivated vaccine. When measuring the virus load in serum and tissues, both vaccines were verified to protect mice from CHIKV infection. Recently, the means of inoculation has also been improved. Rudd et al. (2015) introduced Foroderm for the delivery of inactivated CHIKV vaccine using cylindrical silica microparticles. This needle-free strategy greatly improves the convenience of vaccination.

The stability and safety of inactivated vaccine come at the expense of efficacy and production cost, which, to a certain extent, impedes its accessibility. The development of inactivated vaccines shows a less prosperous trend than vaccines based on other strategies.

## Subunit Vaccine

Subunit vaccine, like inactivated vaccine, is an early mature strategy for vaccine preparation. The viral envelope or capsid is obtained through chemolysis or proteolysis to prepare the vaccine. By using individual viral proteins, subunit vaccines elicit an immune response without inducing the production of antibodies against unrelated antigens or infectious viral particles. This method not only ensures the safety of the vaccine but also makes large-scale manufacturing possible.

The CHIKV envelope glycoproteins E1 and E2 have been selected to develop CHIKV subunit vaccines in different expression systems. Metz et al. (2011) first expressed E1 and E2 in insect cells. Both proteins were N-glycosylated and membrane-targeting, consistent with the maturation of E1 and E2 in whole CHIKV virion. E2 antibody generated by this baculovirus expression system was able to neutralize CHIKV in rabbits. Next, they compared the immunogenicity of three recombinant baculoviruses: E1, E2, and CHIKV VLPs. Although all three baculoviruses induced neutralizing antibodies, the titers of the subunits were not as high as that of VLP. When challenging AG129 mice with infectious CHIKV, VLP protected all animals, while only half of the mice vaccinated by the subunits finally survived. These results suggested that the efficacy of subunit vaccine was not as good as that of other strategies (Metz et al., 2013). This was partially confirmed by other studies that identified the important role of adjuvants in subunit vaccine application (Khan et al., 2012; Kumar et al., 2012). Also, subunits based on CHIKV E1 and E2 were alternatively generated in bacterial expression systems. E1 and E2 proteins from bacteria successfully neutralized CHIKV and completely protected BALB/c mice from disease induced by CHIKV. However, the efficacies varied with different adjuvants, indicating that the immune response to subunit vaccine largely depended on adjuvants.

## LAV

Live-attenuated virus is generated by modifying the structure of a virus to significantly weaken its virulence while retaining its immunogenicity. Compared with inactive vaccine, which is a fully inactive pathogen, LAV can stimulate a stronger and long-term immune response. However, higher immunogenic ability is a trade-off for lower safety. In this regard, the safety of LAV should be cautiously evaluated during animal model and clinical trials (Plante et al., 2015). Owing to its high immunogenic potency, LAV is the platform with the best prospects. It can also be combined with other strategies such as vector vaccine, which further extends its application.

After CHIKV enters cells, early immune response is activated by nsPs. Thus, several LAVs have been designed that target nsPs. By the use of the concept well established in the Semliki Forest virus (SFV), Chan et al. (2019) introduced point mutation to generate three CHIKV mutants. The specific mutation sites and corresponding vaccine candidates were as follows: RH-CHIKV, with R532H substitution in the cleavage region between nsP1 and nsP2; EV-CHIKV, with E515V substitution in nsP2; RHEV-CHIKV, with combinational double mutations of R532H and E515V. Researchers characterized the activity of the three mutants *in vitro* and found that the viral RNA titers of both RH-CHIKV and RHEV-CHIKV were lower than that of WT-CHIKV. Consistent with this result, RH-CHIKV and RHEV-CHIKV induced high levels of IFN-α and IFN-γ. To clarify whether the reduced activity exhibited in mouse tail fibroblasts affected symptoms *in vivo*, WT and mutant CHIKV were injected into adult mice. Acute viremia was largely alleviated in RH-CHIKV and RHEV-CHIKV infected mice. Mutant CHIKV also changed the cytokine pathway toward anti-inflammatory response. When challenged by the closely related O’nyong-nyong virus, no obvious viremia was detected in nsP-mutant-infected mice, indicating broad cross-protection by the vaccines.

Another LAV targeting nsP was Δ5nsP3, in which 180 nucleotides in the replicase region of nsP3 were deleted. Single-dose immunization with Δ5nsP3 generated high titers of neutralizing antibodies that lasted until the day of virus challenge (LR2006-OPY1 strain, day 123). It also induced IFN-γ T-cell responses. By determining the viral load in plasma and measuring hematological parameters, Δ5nsP3 vaccination showed protective efficacy in cynomolgus macaques against CHIKV infection. In this study, researchers evaluated another two LAVs. One was a DNA-launched replicon vaccine, DREP-E, in which the capsid of CHIKV was deleted. The other was a recombinant modified vaccinia virus that encoded the full CHIKV C-E3-E2-6K-E1. Both vaccine candidates showed similar results as Δ5nsP3, indicating good prospects for the future application of LAV (Roques et al., 2017).

Capsid was another potential target for LAV development. Recently, a novel vaccine candidate against CHIKV was generated in which the capsid was completely deleted (Zhang et al., 2019). Researchers found that capsid was dispensable for ΔC-CHIKV virion assembly. Consistent with this, ΔC-CHIKV successfully propagated in BHK-21 cells and had similar antigenic activity to WT-CHIKV. Single-dose vaccination (10^4^ PFU) of C57BL/6 mice and immunocompromised IFNAR^–/–^ mice protected them from subsequent CHIKV (ECSA strain) challenge. ΔC-CHIKV efficiently induced neutralizing antibodies, comparably with WT CHIKV. No footpad swelling was observed in ΔC-CHIKV immunized mice. The attenuated infection in IFNAR^–/–^ mice indicated that ΔC-CHIKV could be a potential vaccine. The researchers specially assessed the stability of ΔC-CHIKV, since it was an important concern for all LAVs. After five passages, no detectable genomic change was reported. The efficacy and safety of ΔC-CHIKV suggested that it is a promising vaccine candidate.

Taylor et al. (2017) found a nuclear localization sequence (NoLS) in the N-terminal region of capsid protein that was important for virus replication and developed a vaccine candidate by site-directed mutagenesis. Mechanistically, they discovered that the attenuated replication resulted from reduced nuclear import of capsid protein. Attenuation was confirmed by measuring the virus copy number in the supernatants of cultured BHK-21 and C6/36 cells. Mice administered a subcutaneous injection of CHIKV-NoLS showed no disease symptoms. Cross-protection was monitored when CHIKV-NoLS-immunized mice were challenged by another alphavirus, Ross River virus (RRV). The attenuation and stability of CHIKV-NoLS were further evaluated by histological and flow cytometric analysis (Abeyratne et al., 2018). Mice inoculated with CHIKV-NoLS exhibited minimal inflammation in the footpad compared with a CHIKV-WT infected group. When stored at −20°C and −80°C for up to 56 days, the titer of CHIKV-NoLS remained stable. Besides, CHIKV-NoLS showed no loss of infectivity after freeze and thaw. These results confirmed the preclinical safety and stability of CHIKV-NoLS.

Recently, a novel genomic rationale was adopted by Carrau et al. (2019). Multiple replacements of synonymous codons were made in the CHIKV genome to reduce the mutational robustness of the virus and led to a deleterious evolutionary direction (Carrau et al., 2019). Synonymous codons that had the highest likelihood of becoming stop codons (“1-to-Stop,” one mutation away from stop) were examined. They constructed two candidates. Specifically, the STOP virus had 151 “1-to-Stop” synonymous codons for all Leu and Ser, while the SuperStop virus had 285 “1-to-Stop” synonymous codons for leucine, serine, arginine, and glycine in the structural-protein-coding region of the CHIKV genome. In experiments on mice, attenuated virus infectivity and diminished disease symptoms were observed. One prominent advantage of this approach was its safety. Since hundreds of synonymous mutations were generated, the reversion risk was greatly reduced.

## Recombinant Virus-Vectored Vaccine

Recombinant virus-vectored vaccine is obtained by inserting genes encoding exogenous protective antigen into the vector virus genome. Recombinant vector vaccine offers the advantages of safety and easy inoculation. A variety of virus vectors, such as poxvirus, herpesvirus, adenovirus, and paramyxovirus, have been used in vaccine development.

Recombinant measles virus vector is widely used to develop CHIKV vaccines and performed well in phase I and II clinical trials. Rossi et al. (2019) tested the immunogenicity and efficacy of their new developed measles virus-vectored vaccine on cynomolgus macaques. Serum was examined by plaque reduction neutralization test and enzyme-linked immunosorbent assay (ELISA). The results indicated that a robust immune response was elicited by measles virus-vectored vaccine. There was no obvious difference in hematology and clinical chemical indicators between control and the vaccinated group. The clinical symptoms of the disease, mainly referring to fever here, were not observed either after vaccination or after virus challenge. Macaques were also protected from viremia. The efficacy and safety of this vaccine were confirmed by the outcome of clinical trials. In a randomized, double-blind Phase I clinical trial, the seroconversion rate was 44–92% with a single dose and reached 100% after a second vaccination (Ramsauer et al., 2015). Recently, a double-blind, randomized, placebo-controlled and active-controlled phase II trial was carried out and reported (Reisinger et al., 2019). Participants aged 18–55 respectively received control vaccine (*n* = 34), MV-CHIK (*n* = 195), or measles prime and MV-CHIK (*n* = 34) by intramuscular injections between August 17, 2016, and May 31, 2017. Neutralizing antibodies specifically against CHIKV were detected in MV-CHIK treatment groups, with no serious adverse events reported. Due to its good safety and immunogenicity, MV-CHIK is a promising vaccine candidate for CHIKV.

Most CHIKV vaccine candidates are delivered through injection subcutaneously at the wrist or intramuscularly in the quadriceps muscles. Taking advantage of their platform based on adenovirus 5, the researchers developed an oral CHIKV vaccine (Dora et al., 2019). Preservation in tablets promoted further processing, facilitated non-sterile packaging, shortened the production time, and reduced the economic cost. Oral administration also alleviated the discomfort of vaccination. A replication-deficient 5 type adenovirus (rAd) with a lack of E1 and E3 allowed for the expression of different antigens concurrently, which made it an ideal platform for vaccine development. The researchers have constructed three vaccine candidates with different combinations of CHIKV structural proteins: Ad-CHIKV-SG (expressing C-E3-E2-6K/TF-E1), Ad-CHIKV-E3/E2/E1, and Ad-CHIKV-E3/E2/6K (Dora et al., 2019). All three vaccines induced high IgG titers against CHIKV at week 7, while the former two candidates showed significantly higher titers than Ad-CHIKV-E3/E2/6K. C57BL/6 mice were adopted to test immunogenicity and protection against CHIKV disease. A single dose of 10^8^ IU administration intranasally gave rise to neutralizing antibodies and protected mice from viremia and footpad swelling.

Another adenovirus-based study utilized replication-deficient chimpanzee adenovirus. Due to the induction of anti-adenovirus immune response, human adenoviral-vectored vaccines are not suitable for humans. Chimpanzee adenovirus overcomes this problem to maintain vaccine efficacy (Lopez-Camacho et al., 2019). In this study, the full length or capsid-deleted structural proteins of CHIKV were expressed at the adenoviral platform to generated vaccines ChAdOx1 sCHIKV and ChAdOx1 sCHIKV ΔC. A single dose of the two kinds of ChAdOx1 vaccines was injected in BALB/c mice. Two weeks after vaccination, mice that had received either of the two vaccines showed a high T-cell response frequency. ChAdOx1 sCHIKV and ChAdOx1 sCHIKV ΔC also induced a robust humoral response, indicated by a high level of CHIKV-specific IgG. The above results also highlighted that ChAdOx1 sCHIKV did not require an adjuvant to achieve efficacy. Having confirmed the immunogenicity of ChAdOx1 sCHIKV, further studies will be needed to promote preclinical trials.

Vectors are also suitable for bivalent vaccine. ZIKV, an enveloped positive-stranded RNA virus, shares similarities with CHIKV in clinical symptoms and transmission route. Chattopadhyay et al. previously developed a CHIKV vaccine based on VSV vector (VSVΔG-CHIKV), in which the G protein of VSV was replaced by CHIKV E3-E2-6K-E1 envelope polyprotein (Chattopadhyay et al., 2013). They went further to additionally express ZIKV envelope glycoproteins on that platform (VSVΔG-CHIKV-ZIKV) (Chattopadhyay et al., 2018). Vaccination with VSVΔG-CHIKV-ZIKV induced neutralizing antibody in immunocompetent BALB/c mice and type-I IFN receptor-deficient A129 mice. The immune response was sufficient to protect immunized animals from ZIKV and CHIKV infection, with no viremia detectable. Additionally, the deletion of the G protein also eliminated the neurotropism of VSV, making it safer and more efficient.

## VLP

Virus-like particle is assembled by expressing viral structural proteins that possess self-assembly capacity. With antigenic epitopes similar to wild-type virus, VLP can induce high neutralizing antibody titer. VLP has high safety due to its deficiency in replicative and infectious ability (DeZure et al., 2016). Although the development of VLP vaccine started later than that of traditional strategies, several prominent VLP vaccine candidates have been developed recently.

Virus-like particle vaccines have shown prospective applications, but the mammalian expression systems in which VLP vaccines have been produced limited productivity and increased cost. *Pichia pastoris*, an ideal platform for protein expression, has been adopted to produce VLP for many viruses. The *Pichia* system not only supplied native circumstance for protein expression but also increased the performance-to-price ratio to a large extent (Vogl et al., 2013). Saraswat et al. (2016) have made a good attempt to utilize a yeast expression system for developing CHIKV VLP vaccine. They successfully introduced the gene expressing CHIKV whole structural proteins into the *Pichia* expression system and confirmed the morphological identity of VLPs with CHIKV by electron microscopy. Next, the potential of this product (CHIK-VLPs) to be a vaccine candidate was extensively evaluated. ELISA and plaque reduction neutralization testing showed that CHIK-VLPs induced high-titered antibodies with super specificity and neutralizing activity. Elevated levels of TNF-α and IL-10 indicated a robust cellular response, which combined with restricted levels of IL-2, IL-4, and IFN-γ to make a balanced response. The humoral and cellular immune response elicited by CHIK-VLPs was consistent with the protection of CHIK-VLP-immunized BALB/c mice against CHIKV pathogenesis.

In another study, researchers evaluated the efficacies of different VLP formulations with or without an adjuvant in protecting adult and aged mice (Arevalo et al., 2019). Although VLP alone was able to protect adult mice against CHIKV disease, the disease was even more serious in aged mice vaccinated by VLP alone or VLP plus QuilA adjuvant. ELISA and microneutralization assays showed that immunization elicited a high level of neutralizing antibody titer specifically against CHIKV in adult mice. However, for aged mice, negligible antibody was detected. This research implied that specific vaccines suitable for the elderly should be developed in the future.

## Chimeric Vaccine

Chimeric vaccine links the genome or genome fragments of at least two pathogens by genetic engineering to express antigens from multiple pathogens simultaneously. The most attractive advantage of chimera vaccines is a high immune response against multiple pathogens. Besides, since it is constructed by genomic methods, chimeric vaccines are more stable than traditional LAV.

The ideal vaccine would balance safety and immunogenicity; low performance in one or the other is the disadvantage of LAV and inactivated vaccine, respectively. To overcome this issue, Erasmus et al. (2017) creatively used an insect-specific alphavirus, Eilat virus (EILV), to contain CHIKV structural proteins. Its deficiency of replication gave this chimera virus a high level of safety. However, the entry and delivery of RNA during the early stage of virus replication resembled that of wild-type CHIKV. They first identified that EILV/CHIKV virus had an identical structure to wild-type CHIKV. They then chose immunocompetent C57BL/6 mice, immunocompromised A129 IFNα/βR^–/–^ mice, and cynomolgus macaques to conduct *in vivo* experiments. A single dose elicited rapid seroconversion 4 days post-vaccination (DPV). Meanwhile, antigen-specific IFN-γ-producing CD8^+^ T cells were induced. Challenged by CHIKV at 30 DPV, all C57BL/6 mice vaccinated by EILV/CHIKV were protected from viremia. For IFNα/βR^–/–^ mice, which were utilized for long-term efficacy assessment, EILV/CHIKV vaccination also protected them from weight loss, footpad swelling, viremia, and death. Finally, EILV/CHIKV vaccination was tolerated in cynomolgus macaques. The body temperature remained at baseline level, and viremia was not detectable after CHIKV challenge. In addition, EILV/CHIKV exhibited cross-neutralization against three different strains from the Asian, West African, and Indian Ocean lineages (Erasmus et al., 2017). Another alphavirus, Sindbis virus (SINV), is commonly used as the genetic backbone for chimeric vaccine and successfully protected cynomolgus macaques against lethal eastern equine encephalitis virus (EEEV). However, the Eilat virus is hosted only by insects, further ensuring the safety of the chimeric vaccine.

## Nucleic Acid Vaccine

DNA vaccine is a novel platform that has been developed in recent years. By introducing exogenous DNA into the host, antigen proteins are synthesized by the host expression system. The obvious advantage of DNA vaccine is its simplicity of production. Additionally, DNA vaccine is stable at low temperature, which makes it convenient to store and transport across long distances (Powers, 2018). However, there are several drawbacks to this strategy. Integrating exogenous DNA may elicit an autoimmune response in the host. The immunogenicity is low in humans, and thus vaccination needs repeated boosters as well as adjuvants (Powers, 2018). Addressing these concerns will enable breakthroughs in DNA vaccine design.

Hidajat et al. have developed a new method called iDNA^®^ infectious clone technology, which generates vaccine from plasmid DNA both *in vitro* and *in vivo* (Hidajat et al., 2016). It is distinct from traditional infectious clone technology, which needed *in vitro* RNA transcription and *in vitro* transfection involving bacteriophage polymerase, in that an iDNA^®^ infectious clone uses a CMV promoter to transcribe genomic RNA from a plasmid in eukaryotic cells (Tretyakova et al., 2013). Taking advantage of this novel technology, they developed a DNA-launching vaccine for CHIKV (pCHIKV-7) that encoded the full-length cDNA of 181/25 vaccine. *In vivo* experiments showed that single-dose vaccination with pCHIKV-7 protected mice from CHIKV disease, with neutralizing antibodies being detectable in all animals (Tretyakova et al., 2014). In 2016, researchers analyzed its genetic stability by next-generation sequencing (NGS) (Hidajat et al., 2016). Illumina HiSeq2000 sequencing revealed that overall pCHIKV-7 was more stable than 181/25. As for E2-12 and E2-82 residues, two previously identified attenuating mutations, the frequencies of reversion in pCHIKV-7 were 0.064 and 0.086%, respectively, much lower than that of 181/25 (0.179 and 0.133%).

Conventional vaccines require a lag phase to allow enough antibody generation, which is not suitable for urgent protective need in response to a virus outbreak. Passive immunotherapy such as monoclonal antibody (mAb) prophylaxis provides effective short-term protection. However, repeated injection is necessary due to the short half-life of immunoglobulins. Muthumani et al. (2016) combined the advantages of a passive antibody and vaccination by an *in vivo* delivery method. DNA encoding the biologically active mAb (dMAb) targeting CHIKV envelope was delivered by plasmid rather than virus vector through electroporation. This strategy also circumvented the risk of inducing an immune response against the vector. Immunizing animals by intramuscular injection of dMAb induced antibodies much more rapidly than conventional vaccination methods. Footpad swelling was not observed in immunized animals challenged by the virus. These dMAbs were able to neutralize diverse CHIKV clinical isolates. In conclusion, this study highlighted the advantages of DNA vaccine, which could be combined with other platforms for vaccine development.

A design based on mRNA is one of the newest strategies for CHIKV vaccine. The vaccine can be designed to deliver mAb just like the DNA vaccine introduced above. Kose et al. (2019) isolated neutralizing human mAbs from the B cells of a survivor of CHIKV infection. They introduced the mAb-encoding sequences into lipid-encapsulated mRNA, which was then delivered by infusion. Among all the mAbs being examined, CHKV-24 showed most prominent inhibitory effect in neutralization assay. *In vivo* experiments revealed that infusion of CHKV-24 succeeded in inducing human IgG in both mice and macaques, the latter of which peaked at 24 h after immunization with the dosage varied from 10.1 to 35.9 μg/mL. Compared with proteins, nucleic acids encoding antibodies are easy to produce and cost less. Another strategy for using an mRNA platform is to instruct host cells to express viral antigens for generating antibodies accordingly. A biotech company named Moderna Therapeutics developed an mRNA CHIKV vaccine (mRNA-1388) and made it through to phase I clinical trial, using engineered mRNA that encoded CHIKV structural polyprotein. With a single dose of this mRNA vaccine, a strong immune response was elicited, and 100% protection was achieved in mice (Goyal et al., 2018). This innovation avoided an immune response against engineered mRNA and ensured sufficient protein synthesis.

## Animal Models

Apart from extensive *ex vivo* studies, *in vivo* experiments are important for developing vaccines. Different animal models have provided platforms for evaluating the efficacy and security of potential CHIKV vaccines. The studies summarized in this paper used cynomolgus macaques and mice of multiple strains involving C57BL/6, BALB/c, and A129.

C57BL/6 mice are immunocompetent animals. Because they are easy to breed and their traits are stable, C57BL/6 mice are widely used to evaluate vaccines. Live-attenuated vaccines evaluated in C57BL/6 mice have included CHIKV-NoLS, RH-CHIKV, EV-CHIKV, RHEV-CHIKV, ΔC-CHIKV, “STOP,” and “SuperStop.” C57BL/6 mice have also been used to assess CHIK-VLP.

BALB/c mice are easy to breed and display little gender difference in body weight. BALB/c mice have been widely employed to develop DNA vaccines and recombinant vector vaccines (ChOdAx1 sCHIKV, ChOdAx1 sCHIKV ΔC, Ad-CHIKV-SG, Ad-CHIKV-E3/E2/6K, and Ad-CHIKV-E3/E2/E1).

Another murine model used in CHIKV infection is A129. Since they lack type I interferon receptors, A129 mice are deficient in the innate immune response. However, their adaptive immunity is retained so that they are tolerant to virus challenging (Couderc et al., 2008). A129 mice have been involved in immunogenicity studies of the EILV/CHIKV, VSVΔG-CHIKV, and CHKV-24 mRNA vaccines.

The cynomolgus macaque, as a non-human primate, is particularly suitable for studying the pathogenesis of viral infection. Compared with murine models, non-human primates share more similarities with humans in physiology, metabolism, and immunity (Labadie et al., 2010), making them highly effective for predicting the efficacy of human treatment. Despite the high cost and complexity of breeding them, macaques remain an irreplaceable model in vaccine development. Non-human primates have been proved to be susceptible to CHIKV infection (Labadie et al., 2010). Studies on LAV vaccine Δ5nsP3, vector vaccine MV-CHIK, mRNA vaccine CHKV-24, and chimeric vaccine EILV/CHIKV have set cynomolgus macaque as an animal model for immunogenicity assessment.

## Conclusion

As a rapidly spreading recurrent infectious disease, CHIKF has attracted wide attention. Vaccination is a powerful means to control epidemic diseases including CHIKF, but there is no commercial vaccine against CHIKV at present. The re-outbreak of CHIKF since 2004 boosted CHIKV vaccine development. Researchers have applied a variety of strategies to develop vaccine candidates, some of which have entered the Phase-I or Phase-II stage of clinical trials and show promising application prospects. In this paper, the latest progress in the development and testing of CHIKV vaccine, especially since 2016, was reviewed. The development strategies, immunogenicity evaluation, and protective efficacy against diseases were introduced; the key information is summarized in Table 1.

**TABLE 1:** Recently developed vaccine candidates against CHIKV.

**Type**	**Vaccine**	**Antigen/target**	**Animal model**	**Dosage**	**Route**	**Results**	**References**
LAV	RH-CHIKV EV-CHIKV RHEV-CHIKV	R532H-nsP1 E515V-nsP2	Adult C57BL/6J mice	10^6^ PFU, single injection	Injection in the metatarsal region of the footpad	RH-CHIKV and RHEV-CHIKV reduced CHIKV infectivity and alleviated viremia and joint symptoms. Cross-protection against ONNV	Chan et al. (2019)
LAV	Δ5nsP3 DREP-E MVA-CE	nsP3 envelope and capsid	Cynomolgus macaques	10^5^ PFU, single injection	Subcutaneous injection in the shoulder and in the upper part of the left wrist	Δ5nsP3 did not induce fever, lymphopenia, or increase in cytokine	Roques et al. (2017)
LAV	ΔC-CHIKV	Capsid deletion	6-week C57BL/6J and IFNAR^–/–^ mice	10^4^ PFU, single injection	Subcutaneous injection	Preserves infectivity in BHK-21 cells. Single-dose injection elicited strong protection against CHIKV in both WT and IFNAR^–/–^ mice, without footpad swelling or weight loss	Zhang et al. (2019)
LAV	CHIKV-NoLS	N-terminal region of capsid protein	21-day C57BL/6 mice	10^4^ PFU, single injection	Subcutaneous injection	No sign of footpad welling, viremia reduced, and expression of proinflammatory factors decreased. Cross-protection against RRV. Vaccine thermo-stable, and minimal inflammation and tissue damage	Taylor et al. (2017); Abeyratne et al. (2018)
LAV	Stop CHIKV SuperStop CHIKV	Multiple synonymous mutations in genome to reduce mutational robustness	5-week C57BL/6 mice	10^4^ PFU, single injection	Footpad injection	No footpad swelling, low viremia, high level of neutralizing antibody	Carrau et al. (2019)
Vector virus	MV-CHIK	Measles-vectored CHIKV structural proteins	18–55-year-old healthy volunteers	5 × 10^4^, 5 × 10^5^ TCID50, twice injection; measle-primed group accepted additional injection at −28 day	Intramuscular injection	Safety and tolerance similar to measle vaccine	Reisinger et al. (2019)
Vector virus	MV-CHIK	Measles-vectored CHIKV structural proteins	Adult cynomolgus macaques	0.35 mL single measle vaccine dose, twice injection at day 0 and day 28	Intramuscular injection in the quadriceps muscles	Viremia low. No signs of disease. Cross-protection against other CHIKV strains	Rossi et al. (2019)
Vector virus	Ad-CHIKV-SG Ad-CHIKV-E3/E2/6K Ad-CHIKV-E3/E2/E1	Structural proteins	6–8-week BALB/c mice 4-week C57BL/6 mice	10^7^ infectious units (BALB/c) 10^8^ infectious units (C57BL/6)	Intranasal administration	Induced high tighter of CHIKV-specific IgG antibodies	Dora et al. (2019)
Vector virus	ChAdOx1 sCHIKV ChAdOx1 sCHIKV ΔC	Structural polyprotein	6–8-week BALB/c mice	10^8^ infectious units with or without adjuvant	Intramuscular injection	High immunogenicity	Lopez-Camacho et al. (2019)
Vector virus	VSVΔG-CHIKV	CHIKV: E3-E2-6K-E1 envelope polyprotein ZIKV: envelope glycoproteins	6–8-week BALB/c mice 7-week A129 mice	10^7^ PFU, single injection	Intramuscular injection	Induced neutralizing antibody responses to both CHIKV and ZIKV. Protected from both CHIKV- and ZIKV-induced diseases	Chattopadhyay et al. (2018)
Chimeric virus	EILV/CHIKV	EILV cDNA clone containing CHIKV structural proteins	4-week C57BL/6 mice; 6-week IFNα/βR^–/–^ mice Cynomolgus macaques	8.8 log10 PFU (C57BL/6) or 8.5 log10 PFU (IFNα/βR^–/–^mice) of live EILV/CHIKV, single injection	Subcutaneous injection	Protected from viremia and footpad swelling when challenged by virus on day 30 post-vaccination. Exhibited long-term efficacy in IFNα/βR^–/–^ mice. For cynomolgus macaques, held normal, baseline diurnal body temperatures 31 DPC	Erasmus et al. (2017)
VLP	CHIK-VLP	C-E proteins	6–8-week (adult), 18–24-month (aged) C57BL/6 mice	30 μg VLPs with different adjuvants Three-time vaccination with or without adjuvants	Intramuscular injection	CHIK VLP without adjuvant elicited immune responses and protected adult mice but exacerbated disease on aged mice	Arevalo et al. (2019)
VLP	CHIK-VLPs	Structural proteins introduced into yeast expression system	4-week BALB/c mice	10, 20, and 40 μg of yeast-derived CHIK-VLPs (in Freund’s adjuvant); boosted at day 14 and day 28	Subcutaneous injection	High level of specific antibody with high neutralization activity. Induced robust humoral and cell-mediated response	Saraswat et al. (2016)
DNA vaccine	dMAb	CHIKV envelope	B6. Cg-Foxn1nu/J mice	100 μg CVM1-Fab plus 25 μg CHIKV Env plasmid injected three times at 2-week intervals	Intramuscular injection	One injection produced antibodies rapidly and neutralized diverse CHIKV clinical isolates	Muthumani et al. (2016)
DNA vaccine	pCHIKV-7	Full-length cDNA of 181/25 vaccine	–	–	–	Genetic stable	Hidajat et al. (2016)
RNA vaccine	CHKV-24	Lipid-encapsulated neutralizing human mAbs	AG129 mice Cynomolgus macaques	10, 2, or 0.4 mg/kg, single injection	Intravenous injection	Protected mice from arthritis, musculoskeletal tissue infection, and lethality and reduced viremia to undetectable levels	Kose et al. (2019)

*LAV, live-attenuated virus; VLP, virus-like particles; –, not mentioned.*

Future studies should pay more attention to the following aspects: (1) Comprehensive consideration of both safety and immunogenicity. This is a common concern for all vaccine production. On the premise of keeping a low rate of revertant mutation of the virus, the vaccine titer should be enhanced to achieve better protection efficacy. (2) Convenience of production and use. Vaccines should be thermostable and easy to produce, transport, and store. In addition, their administration should be convenient, and the discomfort of vaccination should be alleviated. Bivalent and multivalent chimeric vaccines can immunize against more than one virus at one time, which will greatly improve the immune response. (3) Long-term and short-term protection. Vaccines should provide long-term protection. However, acute protection is needed in local areas in times of outbreak. From this point of view, DNA vaccine with both long-term and short-term protective efficacies is a good choice (Muthumani et al., 2016). (4) Different performance in different populations. In one study involving adult and aged mice, vaccines that showed protective efficacies in adult mice exaggerated the symptoms of disease in aged mice (Arevalo et al., 2019). This result suggests that the populations had a significant difference in response to the vaccine. Attention should be paid to the complexity of the social population, especially when determining the dose of vaccine to use. Broadly speaking, most vaccine studies are based on animal models, whether the developed vaccine is suitable for humans has yet to be evaluated. Clinical trials need to be promoted. It is believed that with the progress of new strategies and studies, commercial CHIKV vaccine with high safety, strong immunogenicity, convenient development, and moderate cost can be developed in the near future.

## Author Contributions

LZ and SS provided the concept. SG drafted the manuscript. SG and LZ revised the manuscript. All authors approved the final version for publication.

## Conflict of Interest

The authors declare that the research was conducted in the absence of any commercial or financial relationships that could be construed as a potential conflict of interest.
